# Higher Yogurt Consumption Is Associated With Lower Risk of Colorectal Cancer: A Systematic Review and Meta-Analysis of Observational Studies

**DOI:** 10.3389/fnut.2021.789006

**Published:** 2022-01-03

**Authors:** Jiangjie Sun, Jiangyan Song, Jie Yang, Le Chen, Zuochuan Wang, Meiwen Duan, Shuhui Yang, Chengyang Hu, Qingquan Bi

**Affiliations:** ^1^Health Management College, Anhui Medical University, Hefei, China; ^2^Clinical Medical College, Anhui Medical University, Hefei, China; ^3^College of Nursing, Anhui Medical University, Hefei, China; ^4^School of Humanistic Medicine, Anhui Medical University, Hefei, China

**Keywords:** yogurt, colorectal risk, systematic review, meta-analysis, cohort studies

## Abstract

**Background:** Yogurt is known to be nutrient-rich and probiotic content, which gather optimism due to their potential role in preventing and managing cancers. The effect of yogurt consumption on colorectal cancer (CRC) is inconsistent.

**Objective:** This study aims to investigate the association of yogurt consumption with the risk of CRC.

**Methods:** Three databases, namely, PubMed, Web of Science, and Embase, were searched for all relevant studies from July 2021 on the association of yogurt consumption with CRC risk. We pooled the odds ratios (ORs) and their 95% CIs using a random-effects meta-analysis to assess the association.

**Results:** Finally, 16 studies met the inclusion criteria and were chosen in the meta-analysis. Yogurt consumption was significant with lower risk of CRC risk in the overall comparison (OR = 0.87, 95% CI: 0.81–0.94), in the cohort studies (OR = 0.91, 95% CI: 0.86–0.97), and case-control studies (OR = 0.75, 95% CI: 0.65–0.85). With regard to subgroup analyses by study region, cancer type, publication year, and sex, yogurt consumption significantly decreased overall CRC, colon cancer, and distal colon cancer risks. In stratified analyses, we observed significantly decreased CRC risk in Europe and Africa and published after 2010 and overall population. Sensitivity analysis indicated the result is stable and there is no publication bias in the meta-analysis.

**Conclusions:** Overall, this study indicated that yogurt intake was related to a decreased risk of CRC.

## Introduction

Colorectal cancer (CRC) is the third most common cancer among men and women in the world (1–4). Some known risk factors for the development of CRC have been identified, such as genetic predisposition and epigenetic factors, tobacco use, overweight and obesity, and low physical activity (5–8). Moreover, CRC is also easily influenced by a wide range of dietary factors, such as regular alcohol consumption (9, 10), low fruit and vegetables diet (11–13), low-fiber and high-fat diet, or a diet high in processed meats (14, 15). Over the past decade, a growing number of epidemiological studies have suggested that the gut microbiome builds a unique ecosystem inside the gastrointestinal tract to maintain homeostasis and that gut microbiome compositional changes are highly related to the risk of CRC (16–20). Previous studies have suggested that the equilibrium of gut microbiota is affected by diet factors and any change may create an environment that might foster or prevent tumorigenesis of the intestinal system (21, 22). Thus, the gut microbiota is proposed to play a crucial mediator role in the association of dietary factors with CRC. The gut microbiota is a complex composed of trillions of viruses and microbial cells, which affect many aspects of physiology and human health (23–29).

Fermented food contains a large number of live microorganisms, so it can be used as probiotics to enrich the intestinal tract with beneficial bacteria. It helps the body to absorb nutrients and enhance immune function by preventing inflammation and stimulating phagocytosis (30). Yogurt is one of the representatives and popular fermented foods worldwide, and consumption of yogurt has been reported to associate with a wide range of health benefits in different populations (31–35). The potential mechanisms are complicated, but have been identified as producing immune-modulating metabolites, such as short-chain fatty acids (36); preventing pathogens from entering the intestinal epithelium (37); generating antimicrobial compounds (38); producing proteolytic enzymes (39); reducing the fecal enzyme activity of azoreductase, nitroreductase, and b-glucoronidase, which convert the procarcinogens to carcinogens in the colon (40). Over the past several decades, many epidemiological pieces of evidence have reported that yogurt consumption is associated with decreased risk of metabolic syndrome (41), hip fracture (42), type 2 diabetes (43), cardiovascular diseases (44), etc. However, nutritional information and health-related properties of yogurt in disease progression are limited. Disregarding a growing number of observational studies that have been performed to assess the association of yogurt consumption with CRC risk, the available evidence was inconsistent, several epidemiological studies have indicated an inverse association (45–49), while several other epidemiological studies reported non-significant associations (30, 50–60). More recently, Godos et al. (61) performed an umbrella review of observational studies on the associations of dairy foods with health and reported that yogurt intake may be associated with various health outcomes, yet with too limited evidence to draw definite conclusions. Thus, it is necessary to further clarify the association between yogurt intake and the risk of CRC.

To the best of our knowledge, previous reviews always included the small number of epidemiological studies and did not reach a consensus (62–64). In view of the inconsistent findings in the literature, and lack of a comprehensive systematic review and meta-analysis of the existing literature, an updated systematic review and meta-analysis is needed to further clarify the associations. We performed a meta-analysis of observational studies to clarify the association of yogurt intake with the risk of CRC. Our hypothesis was that higher yogurt intake is associated with a lower risk of CRC.

## Methods

### Protocol and Research Question

This study was presented according to the Preferred Reporting Items for Systematic reviews and Meta-Analyses (PRISMA) 2020 statements (65). We provided the PRISMA checklist in Supplementary Table 1. The participant, exposure, comparison, outcome, and study design (PECOS) are grouped in Supplementary Table 2. The research question of this study is presented as follows: among the general population, is higher yogurt intake related to a lower risk of CRC?

### Data Source and Search Strategy

**Figure 1:**
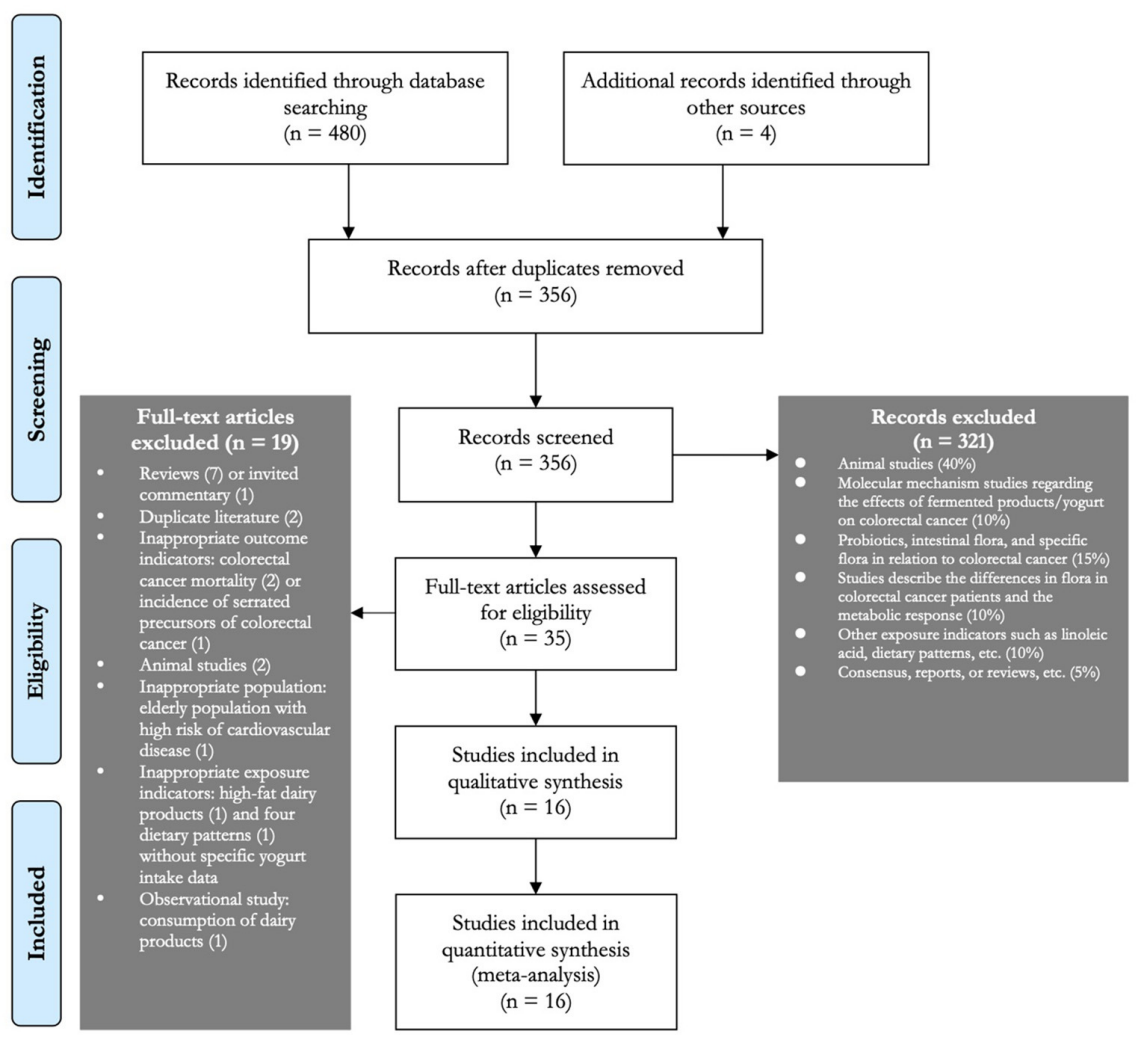
Flow diagram of study selection process.

PubMed, Embase, and Web of Science literature databases were searched dated up to July 2021, using the combinations of keywords related to yogurt and CRC. Keywords for exposure (yogurt consumption) included “yogurt,” “yogurt,” and “cultured milk products,” while keywords for the outcome (risk of CRC) included “colorectal cancer” and “colorectal neoplasms.” The detailed search terms used in each literature database are summarized in Supplementary Table 3. In addition, the reference lists of the chosen studies and any relevant systematic reviews were also checked for any potentially eligible studies not previously identified in this review. Figure 1 depicts the search process.

### Study Eligibility Criteria

The inclusion criteria were (1) human (>18 years old) epidemiological studies (cross-sectional, cohort, or case-control design) that focused on the association of yogurt consumption with an incidence of CRC, such as total CRC, colon or rectal cancer, or proximal or distal colon cancer; (2) studies provided estimates of the odds ratio [OR], relative risk [RR], or hazard ratio [HR] with their 95% CIs for the data synthesis or reported sufficient data that could be used to calculate the estimates was presented; and (3) studies evaluated the intake of yogurt through the use of validated food questionnaires. We excluded studies that (1) were not written in the English language; (2) were not original studies, such as review, meta-analysis, commentary, letter, or editorial; and (4) studies assessed CRC mortality as an outcome of interest.

### Data Extraction

The following data were extracted from each chosen study: name of the first author, year of publication, country, study design (duration of follow-up for cohort studies), sample size, mean age of study participants, dietary assessment, outcome assessment, number of cases, categories of yogurt intake, reported risk estimates (HRs, RRs, or ORs) with their 95% CIs, and the adjusted confounders in the final multivariable regression models. If two effect estimates based on the sex of study participants were reported in a study, we firstly pooled them using fixed-effect meta-analysis and then put the pooled estimate in the main meta-analysis. If studies report the crude and confounding adjusted risk estimates for CRC, we selected the effect estimates from the full-adjusted model.

### Study Quality Evaluation

To assess the quality of each study, we applied the widely used quality assessment tool for an observational study, which is Newcastle-Ottawa Scale (NOS) (66). Two of the authors evaluated the study quality independently using the following criteria: (1) the study selection (maximum 4 points); (2) the adequacy of the outcome in cohort studies and the adequacy of the exposure in case-control studies (maximum 3 points); and (3) the comparability of the studies (maximum 2 points). A study was categorized as high quality if it was assigned with a score ≥7; otherwise, low quality was indicated. Any discrepancies were solved by a group discussion to reach a consensus.

### Meta-Analysis

The reported effect estimates (ORs, RRs, or HRs) were used as the measures of the association of yogurt consumption with the risk of CRC. Following previous practices (67, 68), we considered that standardized risk estimates (e.g., ORs, RRs, and HRs) were equivalent and pooled HRs and RRs with ORs and we used ORs as the indicator of pooled effect size; this is acceptable in the present situation where the outcome is rare (69). To calculate the pooled effect estimates, we compared the highest vs. the lowest categories of yogurt intake, we conducted random-effects or fixed-effect meta-analysis depending on the between-study heterogeneity. When substantial heterogeneity was found, a random-effects meta-analysis was used; otherwise, fixed-effect meta-analysis would be used. The between-study heterogeneity was evaluated using the *I*^2^ statistic (70) and the *P*-value from the Chi-squared test of heterogeneity. We considered an *I*^2^ value ≥50% to indicate substantial heterogeneity and a *P*-value ≤ 0.1 to indicate the presence of statistically significant heterogeneity (71). To test the robustness of the result, sensitivity analysis was performed with the “leave-one-out” method. The potential risk of publication bias was assessed using funnel plot and Egger's test. The sources of heterogeneity were explored by subgroup analyses where available. In the present study, STATA 15.0 (Stata Corp LLC, College Station, TX, USA) was used to perform all analyses.

## Results

A total of 484 studies (PubMed: 108, Web of Science: 248, EMBASE: 124, and other sources: (4) were chosen through the literature search (Figure 1). We excluded 321 papers based on the title/abstract screen, and a brief screening of the full-text article after the duplicated studies (*n* = 128) was excluded. Nineteen studies that did not meet the inclusion criteria were excluded and produced a total of 16 studies were included in the systematic review and meta-analysis (35 studies were detailed assessed). The reference lists of all the 19 studies were also screened, and we found that all the potentially included articles were already chosen. Finally, a total of 16 studies were chosen for the systematic review and meta-analysis.

Table 1 summarizes the main characteristics of the epidemiological studies included in the review. Among the 16 included studies, 9 were cohort studies and 7 were case-control studies. The number of study participants in each study who were ranged from 392 to 477,122 and different kinds of food frequency questionnaires were used to assess the consumption of yogurt, and the ascertainment of cases were always from national or regional cancer registers. Eight studies were performed in Europe, 3 in North American, 2 in Asia, 2 in Africa, and 1 in multiple European countries. Almost all of the included studies adjusted the confounders when investigated the association of yogurt consumption with CRC risk. With regard to the quality assessment, almost all of the included studies were appraised as moderate to high quality (Table 1).

**Table 1:** Characteristics of studies investigated the association of yogurt consumption and colorectal cancer risk.

**Author, year of publication (country)**	**Study design (follow-up, years)**	**Cohort name, sample size and study period**	**Age (mean±SD or range, years)**	**Dietary assessment**	**Outcome assessment**	**Reported risk estimates**	**Adjusted confounders**
Kampman et al. (57) (U.S.)	Case-control (NA)	The HPFS and the NHS cohort studies, 18,398, 1986–1990 and 1980–1988	NA	Semi-quantitative food-frequency questionnaire	Diagnosis of adenocarcinoma polyps of the colon or rectum	HPFS C5 vs. C1: RR = 1.06 (0.72, 1.57) NHS (1984–1988) C5 vs. C1: RR = 0.75 (0.51, 1.11) NHS (1980–1988) C5 vs. C1: RR = 0.89 (0.63, 1.25)	Age, total energy, family history, and saturated fat intake
Kampman et al. (53) (Netherlands)	Cohort study (9)	The Netherlands Cohort Study, 120,852, 1986–1989	55–69	Validated FFQ (150 items)	Record linkage to cancer registries and a nationwide pathology register	64 g/day: Ref 181 mg/day: RR = 1.01 (0.69, 1.48) 287 mg/day: RR = 1.29 (0.89, 1.88) 397 mg/day: RR = 1.18 (0.80, 1.72) 634 mg/day: RR = 1.14 (0.77, 1.68)	Age, gender, family history of colorectal cancer, intake of energy, energy-adjusted intake of fat and dietary fiber, BMI, history of gallbladder surgery
Boutron et al. (58) (France)	Case-control (NA)	NA, 1268, 1985–1990	Cases: 64.2 ± 10.3 Controls: 62.1 ± 11.6	Detailed 2-h questionnaire about the diet in the past year	Registry of Digestive Tumors of Burgundy	Tertile 1: Ref Tertile 2: RR = 1.0 (0.7, 1.7) Tertile 3: RR = 1.0 (0.6, 1.6)	Age, sex and caloric intake
Kearney et al., (59) (U.S.)	Cohort study (6)	The HPFS cohort study, 47,935, 1986–1992	40–75	Validated FFQ (131 items)	Self-reported, then confirmed by hospital records and pathology reports	<1/month: Ref 1–4/month: RR = 0.70 (0.45, 1.09) 2–4/week: RR = 0.81 (0.51, 1.26) 5–7/week: RR = 0.96 (0.51, 1.26) > 1/day: RR = 1.09 (0.70, 1.72)	Age, total calories, family history for colon cancer, previous potyp, screening, past history of smoking, alcohol, aspirin, physical activity, BMI, red meat, saturated fat, and dietary fiber
Jarvinen et al. (54) (Finland)	Cohort study (15)	Population cohort from a large-scale health examination survey performed by the Social Insurance Institution's Mobile Clinic, 9959, 1966–1991	> 15	Performed questionnaire	Linkage to the Finish Cancer Registry	Colon cancer Q4 vs. Q1: RR = 0.79 (0.34, 1.79) Rectum cancer RR = 2.67 (0.91, 7.80) Both cancers RR = 1.28 (0.68, 2.40)	Age, sex, BMI, occupation, geographical area, and intake of energy
Terry et al. (55) (Sweden)	Cohort study (11.3)	Swedish Mammography Screening Cohort, 61,463, 1987–2000	The average age at diagnosis was 67 for colon cancer cases and 68 for rectal cancer cases	FFQ (67 items)	Linkage to regional cancer registry	Colorectal cancer Q4 vs. Q1: RR = 0.90 (0.72, 1.13) Colon cancer RR = 0.76 (0.57, 1.01) Proximal colon cancer RR = 0.67 (0.44, 1.03) Distal colon cancer RR = 0.80 (0.47, 1.35) Rectal cancer RR = 1.28 (0.87, 1.89)	Age, BMI, educational level, total energy, and quartiles of red meat, alcohol, and energy-adjusted folic acid and vitamin C intake
Sanz et al. (49) (Spain)	Case-control (NA)	NA, 392, 1998	Cases: 61.7 ± 10.8 Controls: 61.6 ± 9.8	Questionnaire	Linkage to cancer register	0.97 (0.95, 0.98)	Age, sex and geographical area
Kojima et al. (60) (Japan)	Cohort study (9.9)	Japan Collaborative Cohort Study, 107,824, 1988–1999	40–79	Validated FFQ in Japanese diet (33 items)	The resident registration records of municipalities	Colon cancer: Seldom: Ref 1–2 per month: HR = 1.32 (0.74, 2.35) 1–7 per week: HR = 0.80 (0.42, 1.51) Rectal cancer: Seldom: Ref 1–2 per month: HR = 0.80 (0.39, 1.62) 1–7 per week: HR = 0.46 (0.21, 1.02)	Age, family history of CRC, BMI, frequency of alcohol intake, current smoking status, walking time per day, and educational level and stratified by regions of enrollment
Pala et al. (45) (Italy)	Cohort study (12)	EPIC-Italy cohort, 45,241, 1993–1998	30–86	Three validated semi-quantitative food questionnaires	Linkage of the study cohort to the databases of the regional cancer registries	0–1 g/day: Ref 1–25 g/day: HR = 0.86 (0.65, 1.15) 25–87.5 g/day: HR = 0.65 (0.48, 0.89)	Energy, animal fat, red meat intake, dietary calcium, dietary fiber and simple sugars, BMI, alcohol consumption, smoking, education level, recreational activity, sporting and type of work
Kinany et al. (47) (Morocco)	Case-control (NA)	NA, 2906, 2009–2017	41–71	Validated FFQ (225 items)	Anatomo- pathology reports	CRC ≤44.0 g/day: Ref. > 44.0 g/day: OR = 0.74 (0.64, 0.86) Colon cancer ≤44.0 g/day: Ref. >44.0 g/day: OR = 0.72 (0.58, 0.89) Rectal cancer ≤44.0 g/day: Ref. > 44.0 g/day: OR = 0.76 (0.61, 0.93)	Age in years, residence, education level, monthly income, physical activity intensity, smoking status, BMI categories, NSAIDS, total energy intake, intakes of red processed meat and dietary fiber, family history of CRC
Michels et al. (30) (U.S.)	Cohort study (32)	The NHS and HFPS cohort studies 126,323, 1980–2012 and 1986–2012	40–75	Validated FFQ (61 items and 131 items)	Self-report and then confirmed by medical records and pathology reports	CRC Never or < 1 serving/month: Ref 1–3 servings/month: HR = 0.97 (0.87, 1.07) 1+ servings/week: HR = 0.89 (0.80, 1.00) Colon cancer Never or < 1 serving/month: Ref 1–3 servings/month: HR = 0.97 (0.86, 1.09) 1+ servings/week: HR = 0.87 (0.76, 0.99) Proximal colon cancer Never or < 1 serving/month: Ref 1–3 servings/month: HR = 0.92 (0.79, 1.08) 1+ servings/week: HR = 0.84 (0.70, 0.99) Distal cancer Never or < 1 serving/month: Ref 1–3 servings/month: HR = 1.04 (0.86, 1.25) 1+ servings/week: HR = 0.91 (0.74, 1.12) Rectal cancer Never or < 1 serving/month: Ref 1–3 servings/month: HR = 0.93 (0.75, 1.17) 1+ servings/week: HR = 0.95 (0.76, 1.21)	Age, 2-year follow-up cycle, family history of CRC, history of lower gastrointestinal endoscopy, BMI, height, physical activity, pack-years of smoking before age 30, current multivitamin use, regular aspirin or NSAIDs use, parity in women and age at first birth in women, menopausal status and age at menopause, menopausal status and hormone use in women, total caloric intake, alcohol consumption, and energy-adjusted intake of folate, calcium, vitamin D, total fiber, unprocessed red meat, and processed meat
Negrichi et al. (48) (Algeria)	Case-control (NA)	NA, 400, 2016–2019	55.6 ± 13.0 (control) 55.2 ± 17.0 (case)	Validated FFQ	Medical diagnosed	Rarely: Ref Frequently: OR = 0.63 (0.41, 0.96)	No adjustment was made for multiple testing
Nilsson et al. (56) (Sweden)	Cohort study (30)	Northern Sweden Health and Disease Study, 101,235, 1986–2016	45.9 ± 9.4 (referents) 54.9 ±8.3 (any cancer)	Semi-quantitative FFQ	Linkage to Sweden Cancer Register	Q5 vs. Q1 HR = 0.98 (0.77, 1.25) (men) HR = 0.90 (0.70, 1.15) (women)	Age, screening year, dairy product category, BMI, civil status, education level, physical activity in leisure time, smoking status, recruitment cohort, and quintiles of fruit-and vegetables, alcohol, and energy intake
Barrubés et al. (50) (Spain)	Cohort study (9)	PREvencion con DIeta MEDiterranea study, 7216, 2003–2012	55–80	Validated FFQ (137 items)	Medical records	8 (1–22) g/day: Ref 65 (54–85) g/day: HR = 1.15 (0.70, 1.90) 128 (122–186) g/day: HR = 0.94 (0.56, 1.59)	Intervention group, sex, age, leisure time physical activity, BMI, current smoker, former smoker, never smoker, family history of cancer, education level, history of diabetes and use of aspirin at baseline, tertiles of cumulative average consumption during the follow-up of vegetables, fruits, legumes, cereals, fish, meat, olive oil and nuts (all in g/day) and alcohol (g/day and quadratic term)
Tayyem et al. (51) (Jordan)	Case-control (NA)	NA, 501, 2010–2012	≥ 18	Validated Arabic FFQ (30 items)	Face-to-face interview	Rarely: Ref. Monthly: OR = 1.06 (0.31, 3.62) Weekly: OR = 0.82 (0.29, 2.32) Daily: OR = 0.76 (0.25, 2.32)	Age, sex, total energy, physical activity, smoking, education level, marital status, work, income, other health problems and CRC history
Murphy et al. (46) (Europe)	Cohort study (11)	EPIC, 477,122 (8), 1992–2010	≥ 35	Diet and lifestyle questionnaires	Population cancer registries, kin health insurance records, cancer and pathology registries	CRC ≥ 109 g/day vs. 0 g/day: HR = 0.90 (0.81, 0.99) All colon cancer ≥ 109 g/day vs. 0 g/day: HR = 0.88 (0.77, 1.00) Proximal ≥ 109 g/day vs. 0 g/day: HR = 0.94 (0.79, 1.13) Distal ≥ 109 g/day vs. 0 g/day: HR = 0.84 (069, 1.02) Rectal cancer ≥ 109 g/day vs. 0 g/day: HR = 0.93 (0.79, 1.10)	Total energy intake, body mass index, physical activity index, smoking status and intensity, education status, ever use of contraceptive pill, ever use of menopausal hormone therapy, menopausal status, alcohol consumption and intakes of red and processed meat and fiber, and stratified by age, sex and center

*HPFS, Health Professionals Follow-up Study; NHS, Nurses' Health Study; EPIC, the EuropeanProspective Investigation into Cancer and Nutrition; SD, standardized deviation; OR, odds ratio; RR, relative risk; HR, hazard ratio; BMI, body mass index; FFQ, food frequency questionnaire; CRC, colorectal cancer; TCPS, Tennessee colorectal polyp study; U.S., United States; NSAIDS, non-steroidal anti-inflammatory drugs; HRT, hormone replacement therapy; NA, not available*.

**Figure 2:**
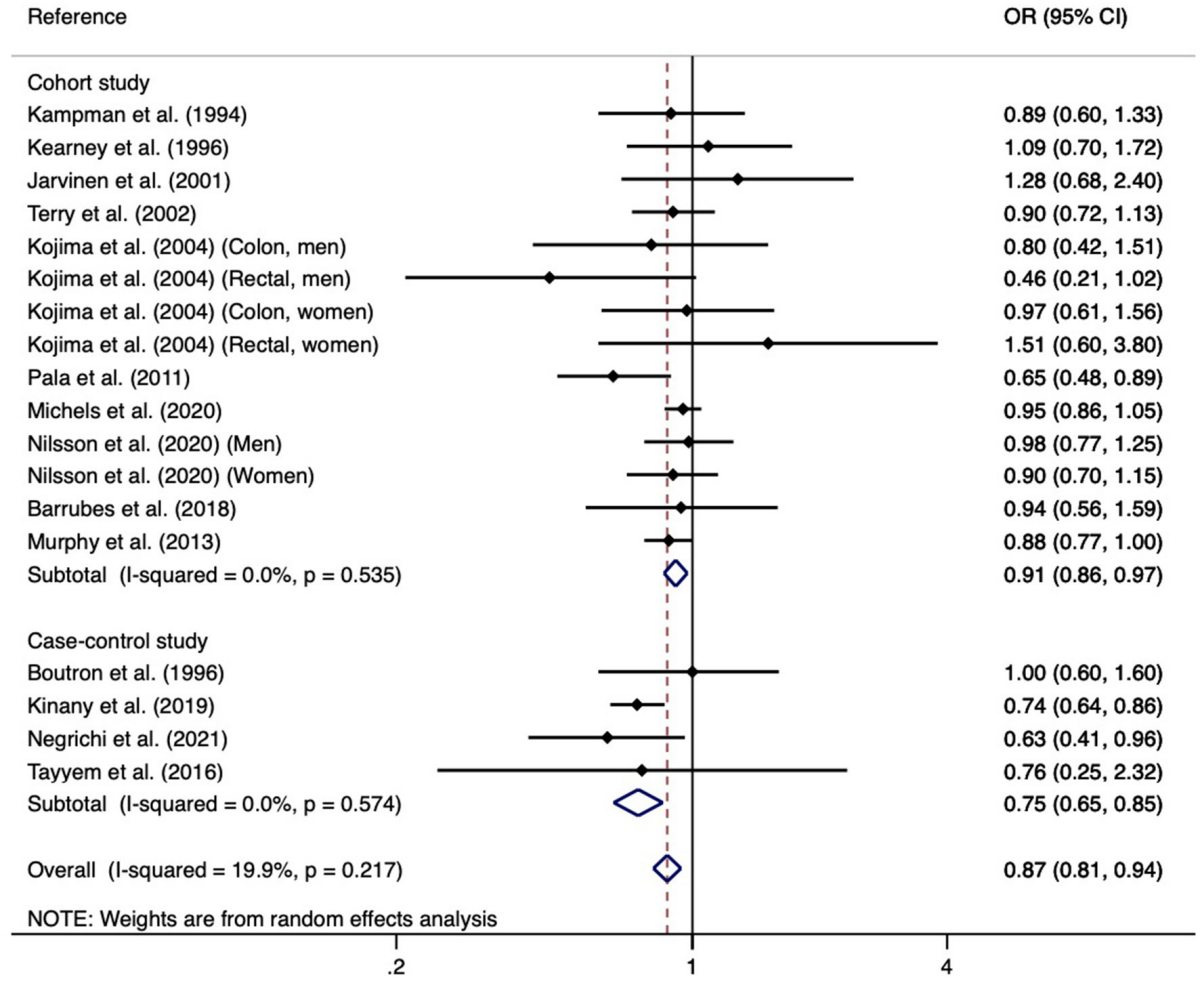
Forest plot of the association between yogurt consumption and risk of colorectal cancer.

**Figure 3:**
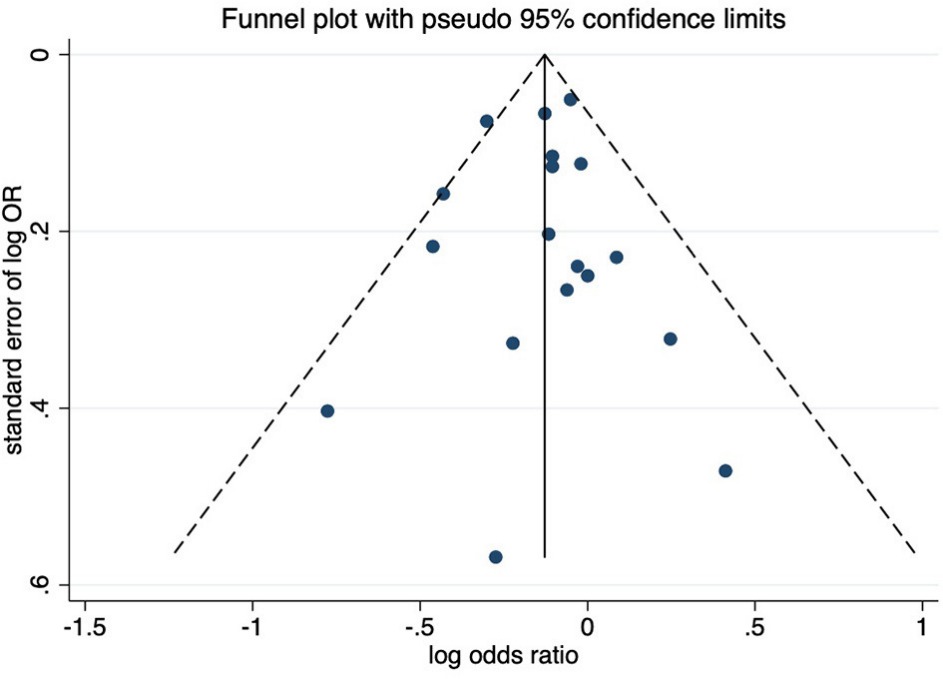
Funnel plot of the association between yogurt consumption and risk of colorectal cancer.

In the meta-analysis, we have found that higher yogurt intake was associated with a lower risk of CRC (pooled OR for the highest compared with the lowest consumption groups: 0.87; 95% CI: 0.81, 0.94; Figure 2). There was no substantial heterogeneity between studies (*I*^2^ = 19.9%; *P*-heterogeneity = 0.217). When performed stratified meta-analyses (Table 2), there is a stronger positive association for case-control studies than in cohort studies (OR = 0.75, 95% CI: 0.65, 0.85 vs. OR = 0.91, 95% CI: 0.86, 0.97). Subgroup analysis by sex indicated no significant associations of yogurt consumption with the risk of CRC in any specific subpopulations. When stratified by publication year, only studies published after 2010 indicated a significant association. When studies restricted to the exposure as fermented milk included yogurt, there is also no significant association with CRC risk. Subgroup analyses by CRC subtype and geographic location revealed significant associations in overall CRC, colon, distal colon, Europe, and Africa. In the sensitivity analysis, each individual study was omitted at a time that did not change the summary effect estimate substantially and the pooled ORs ranged from 0.79 to 0.96. We further excluded one study that has some overlap data, the result was also not changed substantially. The funnel plot in combination with Egger's test for asymmetry (*p*-value = 0.820) did not indicate the presence of publication bias (Figure 3).

**Table 2:** Subgroup analysis of studies investigated the association of yogurt consumption with risk of colorectal cancer.

**Subgroup factors**	***n* of studies**	**OR (95% CI)**	***I*^2^, %**
**Study design**			
Cohort study	10	0.91 (0.86, 0.97)	0
Case-control study	4	0.75 (0.65, 0.85)	0
**Study region**			
Asian	2	0.85 (0.62, 1.17)	4.4
Europe	8	0.89 (0.82, 0.97)	0
Africa	2	0.73 (0.63, 0.84)	0
North American	2	0.96 (0.87, 1.05)	0
**Cancer subtype**			
Colorectal cancer	12	0.87 (0.80, 0.94)	25.8
Colon	6	0.86 (0.78, 0.96)	6.0
Rectal	6	0.95 (0.78, 1.16)	57.6
Proximal colon	3	0.91 (0.81, 1.03)	8.7
Distal colon	3	0.87 (0.77, 0.99)	0
**Yogurt solely**			
Yes	9	0.83 (0.74, 0.93)	38.1
No	5	0.94 (0.84, 1.07)	0
**Publication year**			
Before 2010	6	0.93 (0.81, 1.08)	0
After 2010	8	0.85 (0.77, 0.94)	44.5
**Sex**			
All	10	0.84 (0.76, 0.94)	40.6
Men	4	0.77 (0.55, 1.08)	58.4
Women	4	0.89 (0.77, 1.02)	0

## Discussion

The present meta-analysis identified 16 studies that included a total of 1,129,035 participants. When compared with the lowest category of yogurt intake, the highest category of yogurt consumption was associated with a lower risk of CRC. Importantly, yogurt intake was related to a decreased risk of CRC in both case-control and cohort studies. The effect was more pronounced in case-control studies than in cohort studies. The conclusion of this study is generally in line with evidence from previous meta-analyses that suggested an inverse association of yogurt consumption with the risk of other diseases (33, 43, 72–75).

Over the past few years, the beneficial effects of yogurt consumption on lowing risk of CRC have been supported by a growing number of human epidemiological studies (30, 45, 46, 51, 52, 62). Although the findings were inconsistent, several clinical and epidemiological studies have indicated the important role of yogurt intake in managing weight (76–78). Obesity is a well-known risk factor of CRC (79–81); thus, the above studies indirectly support the beneficial role of yogurt intake in decreasing the risk of CRC. Furthermore, regular yogurt consumption is a good habit and thus may also be associated with decreasing the risk of CRC. This study is in agreement with two previous systematic reviews and meta-analyses that reported yogurt consumption was associated with decreased risk of CRC (62, 63). However, this study has updated the available evidence and is more comprehensive (Table 3). The non-significant association reported from previous original studies can be attributed to the following factors: (1) the definition of exposure is not precise (yogurt has different associations with other food items), and number of living bacteria in the yogurt could also have reduced the power to elaborate the association between the two of previous studies; (2) there are different types of methods used by fermentation processes in different regions, depending on the starter organisms used. The obtained varied yogurt types might give different effects to the results of previous epidemiological studies; and (3) few studies have classified the subtypes of CRC, and yogurt consumption may exert different effects on each subtype of CRC. A possible explanation for the differences in associations between yogurt consumption and CRC risk by different subgroups is that the number of included studies might influence the results. For example, almost half of the included studies (*n* = 8) were conducted in European countries and the dairy products consumption varies greatly among different regions. Europe is the region with the highest dairy products consumption (82). The possible reason for the studies that were published after 2010 showed significant association is that the follow-up durations of the included studies were long enough for the outcome to occur. An only significant association was found for the overall population that has also been reported in the study of Pala et al. (45), the possible reason is that most of the included studies were insufficiently powered to detect a supposed small difference between women and men regarding the protective effect.

**Table 3:** The comparison of protocols between previous systematic reviews and our systematic review.

	**Barrubés et al. (63)**	**Zhang et al. (62)**	**Our systematic review**	**Observation**
**Protocol**				
Databases	MEDLINE(PubMed), Cochrane Library, CINAHL, and ScienceDirect	PubMed, Embase and CNKI	Web of Science, PubMed, and EMBASE	–
Keywords	Dairy products (i.e., “dairy” or “dairy products”) and subtypes of dairy products (i.e., “milk” or “yogurt” or “yogurt” or “cheese” or “cultured milk products”) in combination with keywords related to CRC events (i.e., “colorectal cancer” or “colorectal neoplasms”)	“Fermented food or cheese or fermented milk or cultured milk or yogurt or lactic acid bacteria” and “cancer”	“Yogurt,” “yogurt” and “cultured milk products” in combination with “colorectal cancer” and “colorectal neoplasms”	The keywords of Zhang et al. (62) also focused on other cancers, but Barrubés et al., (63) and our study only focused on CRC. Furthermore, the two previous studies also focused on other dairy products
Searching period Guideline	4 June, 2018 Cochrane Handbook for Systematic Reviews of Interventions MOOSE PRISMA	Before July 2018 Not reported	Before July, 2021 PRISMA	Our study included 7 additional studies due to the updated search; PRISMA guideline is recommended for systematic reviews
Exposure of interest	Total dairy products High-fat dairy products Low-fat dairy products Total milk Whole milk Low-fat milk Fermented dairy products Total yogurt Cultured milk Cheese	Yogurt Cheese	Yogurt	Our exposure analysis is more specific
Outcome of interest for meta-analysis	CRC Colon cancer Colon cancer by site (proximal or distal colon) Rectal cancer	Cancers	CRC Colon cancer Colon cancer by site (proximal or distal colon) Rectal cancer	No difference, all the three studies have assessed CRC
Exclusion criteria	Not report	Not report	Articles does not our inclusion criteria were excluded	–
Types of studies	Case-control and prospective cohort studies	Cohort study or case–control study that published in English language	Epidemiological studies with cohort, cross-sectional, or case-control designs	–
Quality assessment	NOS	None	NOS	NOS is widely used to assess the quality of cohort and case-control studies
Number of included studies	29 studies Yogurt: 7 studies	61 studies Yogurt and CRC: 9 studies	16 studies	Our study included more studies
Statistical analysis Subgroup	Not reported; Study design CRC subsite	Fixed-effects model or random-effects model Study design	Random-effects or fixed-effect meta-analysis	–
Test of heterogeneity	Q test I^2^ statistic	Q test I^2^ statistic	Q test I^2^ statistic	Q test and I^2^ statistic are valid test for heterogeneity
Sensitivity analysis	None	Leave-one-out method	Leave-one-out method	To observe the robustness of pooled analysis, sensitivity analysis is recommended
Publication bias	None	Funnel plot Begg's test	Funnel plot Egger's test	To assess the publication bias, funnel plot and Egger's test are recommended by the Cochrane handbook
Main findings	Yogurt consumption is associated with lower risk of CRC in cohort studies, but not in case-control studies	Yogurt consumption was significantly with decreased CRC risk	Yogurt consumption was significantly with decreased CRC risk	Our study provided more information due to the available of subgroup analyses

*Aune et al. (64) assessed the associations of dairy products with colorectal cancer using systematic review and meta-analysis, but only included two studies and reported an estimate of 1.00 (95% CI: 0.67, 1.48) was thus not compared with our study*.

*PRISMA, the Preferred Reporting Items for Systematic Reviews and Meta-analyses; NOS, Newcastle-Ottawa Scale; CRC, colorectal cancer*.

For a long time, people have believed that yogurt and other fermented dairy products are beneficial to the health of the gastrointestinal tract. Therefore, several pathogenic mechanisms that may have a protective effect on CRC have been proposed. Yogurt can exert anti-tumor effects by reducing the level of carcinogens in the intestine, for example, by reducing the activity of intestinal enzymes, such as nitro reductase and fecal bacterial enzymes, and reducing the level of soluble fecal bile acids, all of which are related to colon carcinogenesis (83, 84). *Lactobacillus bulgaricus* (*L. bulgaricus*) has been shown to prevent tumor induction caused by 1,2-dimethylhydrazine in mouse models (85), and both streptococcus thermophilus and lactobacillus delbrueckii subsp. bulgaricus produce antigenotoxic metabolites that act as blocking agents to prevent initiation carcinogenesis (86).

Compared with previous systematic and meta-analyses focused on the association of fermented dairy foods intake and risk of cancer (62), this is the first meta-analysis that further performed the stratified analyses. All the included studies are appraised as moderate to high quality and evidence from the present meta-analysis is reliable.

Several strengthens should be acknowledged for this study. To our knowledge, this is the first meta-analysis to investigate the association of yogurt intake with risks of CRC and its different subtypes. Moreover, the robustness of the results was tested by performing some sensitivity analyses, and the potential risk of publication bias was also evaluated. Disregarding the strengthens of this study, some limitations should be acknowledged as (1) the number of included studies is relatively small and thus precluded us perform meta-regression analysis to explore source(s) of heterogeneity. Moreover, we only included studies published in the English language so that some other language papers may be omitted; (2) we are unable to explore the dose-response curve of yogurt consumption with CRC risk due to the limited data provided by the included studies; (3) most of the included studies did not distinguish colon and rectal cancers and analyzed them together. In spite of these cancers are always considered together, potential etiological factors for colon and rectal cancers may be different and site-specific mechanisms of carcinogenesis have been indicated (87); (4) although most of the included studies have controlled some important confounders, other potential unmeasured confounders cannot be ruled out and thus influence the results of the meta-analysis; (5) most of the chosen studies were performed in developed countries and thus prohibited us to generalize the results to other countries. Considering that the consumption and making methods of yogurt vary greatly from country to country (88, 89), region-difference should be considered in future studies; (6) the findings were sourced from observational studies and thus cannot establish the causal relationship.

## Conclusion

To conclude, this systematic review and meta-analysis suggested that yogurt consumption is related to a lower risk of CRC. However, in consideration of the aforementioned limitation, these findings should be confirmed by further longitudinal studies with improved yogurt consumption assessment, better CRC, such as subtypes of CRC case ascertainment and comprehensive control of confounders in clarifying the association. If such a conclusion is supported, we would recommend regular yogurt intake as a healthy lifestyle behavior in decreasing the risk of CRC in adults.

## Data Availability Statement

The original contributions presented in the study are included in the article/Supplementary Material, further inquiries can be directed to the corresponding author/s.

## Author Contributions

JSu and JSo conceived the idea, performed the statistical analysis, and drafted this meta-analysis. JY, LC, and ZW selected and retrieved relevant papers. MD and SY assessed each study. JSu was the guarantor of the overall content. CH and QB supervised the whole study process and contributed to the critical revision of the manuscript. All authors revised and approved the final manuscript.

## Funding

This study was supported by Anhui Province Natural Science Foundation (1908085MG233), Quality Engineering for Research Projects of the Anhui Province (2020wyxm108, 2020SJJXSFK1341), and Key Projects of Natural Science Research of Anhui Provincial Department of Education (KJ2020A0163).

## Conflict of Interest

The authors declare that the research was conducted in the absence of any commercial or financial relationships that could be construed as a potential conflict of interest.

## Publisher's Note

All claims expressed in this article are solely those of the authors and do not necessarily represent those of their affiliated organizations, or those of the publisher, the editors and the reviewers. Any product that may be evaluated in this article, or claim that may be made by its manufacturer, is not guaranteed or endorsed by the publisher.
